# *TERT* Promoter Mutations and rs2853669 Polymorphism: Useful Markers for Clinical Outcome Stratification of Patients With Oral Cavity Squamous Cell Carcinoma

**DOI:** 10.3389/fonc.2021.782658

**Published:** 2021-11-10

**Authors:** Silvia Giunco, Paolo Boscolo-Rizzo, Enrica Rampazzo, Giancarlo Tirelli, Lara Alessandrini, Roberto Di Carlo, Marco Rossi, Piero Nicolai, Anna Menegaldo, Valentina Carraro, Margherita Tofanelli, Luigia Bandolin, Giacomo Spinato, Enzo Emanuelli, Monica Mantovani, Marco Stellin, Rossana Bussani, Angelo Paolo Dei Tos, Maria Guido, Marzia Morello, Jonathan Fussey, Giovanni Esposito, Jerry Polesel, Anita De Rossi

**Affiliations:** ^1^ Department of Surgery, Oncology and Gastroenterology, Section of Oncology and Immunology, University of Padova, Padova, Italy; ^2^ Immunology and Diagnostic Molecular Oncology Unit, Veneto Institute of Oncology (IOV), Istituto di Ricovero e Cura a Carattere Scientifico (IRCCS), Padova, Italy; ^3^ Department of Neurosciences, Section of Otolaryngology, University of Padova, Treviso, Italy; ^4^ Department of Medical, Surgical and Health Sciences, Section of Otolaryngology, University of Trieste, Trieste, Italy; ^5^ Department of Medicine (DIMED), Section of Pathology, University of Padova, Padova, Italy; ^6^ Department of Neurosciences, Section of Otolaryngology, University of Padova, Padova, Italy; ^7^ Unit of Oral and Maxillofacial Surgery, Treviso Regional Hospital, Treviso, Italy; ^8^ Department of Medical, Surgical and Health Sciences, Section of Pathology, University of Trieste, Trieste, Italy; ^9^ Department of Medicine (DIMED), Section of Pathology, University of Padova, Treviso, Italy; ^10^ Department of ENT/Head and Neck Surgery, Queen Elizabeth University Hospital Birmingham, Birmingham, United Kingdom; ^11^ Unit of Cancer Epidemiology, Centro di Riferimento Oncologico di Aviano (CRO) Istituto di Ricovero e Cura a Carattere Scientifico (IRCCS), Aviano, Italy

**Keywords:** oral cavity squamous cell carcinoma (OCSCC), telomerase, *TERT* promoter mutations, SNP rs2853669, telomere, prognostic biomarkers, survival

## Abstract

**Objective:**

To date, no useful prognostic biomarker exists for patients with oral squamous cell carcinoma (OCSCC), a tumour with uncertain biological behaviour and subsequent unpredictable clinical course. We aim to investigate the prognostic significance of two recurrent somatic mutations (-124 C>T and -146 C>T) within the promoter of telomerase reverse transcriptase (*TERT*) gene and the impact of TERT single nucleotide polymorphism (SNP) rs2853669 in patients surgically treated for OCSCC.

**Methods:**

The genetic frequencies of rs2853669, -124 C>T and -146 C>T as well as the telomere length were investigated in 144 tumours and 57 normal adjacent mucosal (AM) specimens from OCSCC patients.

**Results:**

Forty-five tumours harboured *TERT* promoter mutations (31.3%), with -124 C>T and -146 C>T accounting for 64.4% and 35.6% of the alterations respectively. Patients with -124 C>T *TERT* promoter mutated tumours had the shortest telomeres in the AM (p=0.016) and showed higher risk of local recurrence (hazard ratio [HR]:2.75, p=0.0143), death (HR:2.71, p=0.0079) and disease progression (HR:2.71, p=0.0024) with the effect being potentiated by the co-occurrence of T/T genotype of rs2853669.

**Conclusion:**

-124 C>T *TERT* promoter mutation as well as the T/T genotype of the rs2853669 SNP are attractive independent prognostic biomarkers in patients surgically treated for OCSCC, with the coexistence of these genetic variants showing a synergistic impact on the aggressiveness of the disease.

## Introduction

With a worldwide estimated age-standardized incidence rate of 4.0 per 100,000 and an estimated number of new cases in 2018 of 354,864, oral cavity squamous cell carcinoma (OCSCC) is the most common carcinoma developing from the epithelial lining of the upper aero-digestive tract (UADT), thus representing an important burden on health care (1).

Based on the histopathological stage, OCSCC can exhibit an unpredictable behaviour with a fraction of patients with early-stage cancer suffering from poor prognosis (2). Patients curatively treated for OCSCC have indeed a high propensity to develop both recurrences and second field tumours (3). Thus, despite recent improvements in the management strategies of OCSCC, improvements in outcomes have been modest (4).

High-risk human papillomaviruses (HPVs), responsible for more than 50% of oropharyngeal squamous cell carcinomas (SCCs) and robust prognostic biomarkers in risk-stratifying in individuals with these malignancies (5), play only a marginal role in OCSCC (6). Thus, since not all OCSCCs are attributable to tobacco and alcohol exposure, the aetiopathogenesis of these neoplasms remains unknown in several cases and no reliable biomarker capable of stratifying the prognosis of OCSCC exists. It is, therefore, of paramount importance to identify biomarkers and molecular signatures predicting cancer relapse that may guide surveillance follow-up strategies and adjuvant treatments.

The infinite proliferation of malignant cells is a hallmark of oncogenesis and telomere/telomerase interplay dictates cell replicative capacity. Telomerase is indeed usually repressed in normal somatic cells, but it is detectable in the vast majority of tumours (7, 8). By synthesizing the telomere sequences and thus preventing cell senescence and apoptosis, the inappropriate activation of the catalytic component of the telomerase, telomerase reverse transcriptase (TERT), appears crucial for maintaining cellular replicative capacity and allowing tumour formation (9). Furthermore, through its non-canonical extra-telomeric functions, the re-activation of telomerase in cancer cells may affect cancer progression and metastasis (10, 11). These properties make TERT a potentially attractive biomarker in cancer.

Among the different mechanisms leading to the inappropriate reactivation of TERT in cancer, mutually exclusive recurrent C-to-T transitions at nucleotides 1,295,228 (-124 C>T) and 1,295,250 (-146 C>T) within the core promoter of *TERT* creating *de novo* binding sites for E-twenty-six (ETS) transcription factors and leading to increased *TERT* gene expression are particularly interesting: first, their prognostic role was consistently observed in several cancers (12), second, among SCCs of the UADT, *TERT* promoter mutations were observed to be topographically restricted to OCSCC (13), and third, unlike assessing *TERT* mRNA levels, *TERT* promoter mutations can be more easily analysed in formalin-fixed paraffin-embedded (FFPE) specimens from routinely collected biopsies.

Although a previous investigation conducted in a population of subject with OCSCC from Taiwan found that those harbouring the -124 C>T *TERT* promoter mutation had a worse prognosis, this was not statistically significant. However, current evidence suggests that the effect of *TERT* promoter mutations may be affected by a common single nucleotide polymorphism (SNP), rs2853669, within the *TERT* core promoter close to the hotspot mutation sites (14). The minor C-variant allele of the SNP disrupts a pre-existing ETS2 binding site at -245 bp in the *TERT* promoter region resulting in decreased TERT expression (15) and thus, counteracts the transactivation activity of the *TERT* promoter hotspots (14). A meta-analysis reports that among cancer patients with *TERT* promoter mutations, the rs2853669 T/T genotype confers a worse prognosis (16), but the modifying role of this SNP in the prognostic value of *TERT* promoter mutations is still controversial (12, 17–20). To date, the prognostic value of rs2853669 in OCSCC remains to be elucidated.

Thus, the main aims of this study were to investigate the prevalence and the clinical significance of *TERT* promoter mutations and the impact of the *TERT* rs2853669 SNP in a larger series of patients surgically treated for OCSCC.

## Materials and Methods

### Patients and Tissue Samples

This is a multi-centre retrospective observational study conducted with the approval of the ethics committee of Treviso/Belluno provinces (Ethic vote: 346/AULSS9) and was performed in a cohort of 144 consecutive patients diagnosed with OCSCC from February 1, 2010 to September 30, 2018, who underwent up-front surgery with/without adjuvant (chemo)radiotherapy, whose samples were available for analysis. All patients gave their informed consent. The study network included three University Hospitals in Northeast Italy, located in Padova, Treviso, and Trieste.

Patients were routinely followed-up [median follow-up time: 43 months; interquartile range (IQR), 28-75 months] according to consensus guidelines with endoscopic examination of the upper aero-digestive tract every 1–3 months for the first year, 3–4 months during the second year, 4–6 months during the 3rd year, and every 6 months thereafter. A dedicated CT scan of the chest was performed annually. Additional dedicated head and neck imaging was arranged based on clinical features and local protocol.

Data for 27 OCSCC samples (tumour tissue, adjacent mucosa and patient characteristics) were available from our previous study (13). One hundred and seventeen specimens were FFPE. Estimations of tumour cell content on FFPE OCSCC sections were made by a trained pathologist. When macrodissection was necessary for enrichment in neoplastic cells, the pathologist marked tumour areas on haematoxylin and eosin-stained tissue slides; the corresponding areas were scraped from four to five serial FFPE sections of 10 μm thickness. Adjacent mucosa from 30 of 117 FFPE specimens was analysed in samples from tumours with negative/clear margins, and the stroma immediately adjacent to the neoplastic epithelium was left as a border zone. DNA from FFPE specimens was extracted using the QIAmp DNA mini kit (Qiagen, Hilden, Germany) according to the manufacturer’s instructions.

### *TERT* Promoter Analysis and Telomere Length Measurement

Genomic DNA amplification for *TERT* promoter region (260 bp) containing -124 C>T and -146 C>T mutation sites, as well as the SNP rs2853669 (-245 T>C), was performed exactly as previously described (21). The amplified products were purified with the Illustra ExoProStar (GE Healthcare, Buckinghamshire, UK) and sequenced on a 3730xl DNA analyzer (Applied Biosystems, Foster City, CA, USA). All samples were analysed in forward and reverse directions.

Telomere length was determined by multiplex PCR assay as previously described (22). Relative telomere length (RTL) values were calculated as telomere/single-copy gene ratio, as previously described (23).

### Statistical Analysis

Differences in socio-demographic and clinical characteristics according to *TERT* promoter were tested through Fisher’s exact test. For each patient, person-time at risk was computed from the date of diagnosis to the event date or the date of last follow-up, whichever came first. Events were defined as death for overall survival (OS), death or recurrence at any site for progression-free survival (PFS), local recurrence for mucosal control, and lymph node recurrence for regional failure. Analyses were truncated at 5 years. The association between *TERT* promoter and oncological outcomes was evaluated using the Kaplan-Meier method, and difference in survival probabilities was evaluated using the log-rank test (24). To account for competing risks, mucosal and regional control were evaluated using cumulative incidence, and differences according to strata were tested using Gray’s test (25). The risk of unfavourable oncological outcome was evaluated using the Cox proportional hazards model (24); multivariable hazard ratios (HR), and corresponding 95% confidence intervals (CI), were calculated adjusting for gender, age, pathological lymph node status (pN), grading, surgical margins, and extracapsular invasion. For mucosal and regional control, HR were adjusted for competing risk according to Fine-Gray model (25).

## Results

### Demographic and Clinical Characteristics of Patients

The clinical characteristics of the patients are summarized in **Table 1**. Globally, the study group had a median age of 65 years (IQR, 54-74 years) at presentation and included 81 (56.2%) male and 63 (43.8%) female patients (**Table 1**). The majority of patients were ever smoking (61.8%) and never drinking (58.3%). Tumour sub-sites within the oral cavity were as follows: 54.2% (78/144) in the tongue, 15.3% (22/144) in the floor of the mouth, 10.4% (15/144) in both the gingiva and the buccal mucosa, and 9.7% (14/144) in other sub-sites including the lip, the hard palate and the retromolar trigone. Pathological stage was T1-T2 in 99 cases (68.7%) and T3-T4 in 45 (31.3%); 47 (32.6%) of the cases had clinically positive regional lymph nodes and 97 cases (67.4%) were N0; collectively, 69 (47.9%) had advanced disease at diagnosis. Nearly 75% of tumours (104/139) showed G1-G2 grading and 25.2% (35/139) were G3. Close/positive surgical margins and positive extra-capsular spread were present in 21 (14.6%) and 16 (11.1%) cases, respectively (**Table 1**).

**Table 1:** Distribution of 144 patients with oral cavity squamous cell carcinoma (OCSCC) according to socio-demographic and clinical characteristics, by *TERT* promoter mutational status.

		*TERT* promoter			Fisher exact test
		unmutated	-124 C>T	-146 C>T	
	N (%)	N (%)	N (%)	N (%)	
Overall	144	99 (68.8)	29 (20.1)	16 (11.1)	
Sex					
Female	63 (43.8)	41 (65.1)	12 (19.1)	10 (15.9)	p=0.3198
Male	81 (56.2)	58 (71.6)	17 (21.0)	6 (7.4)	
Age (years)					
<60	54 (37.5)	43 (79.6)	8 (14.8)	3 (5.6)	p=0.1415
60-69	39 (27.1)	27 (69.2)	8 (20.5)	4 (10.3)	
≥70	51 (35.4)	29 (56.9)	13 (25.5)	9 (17.7)	
Smoking status					
Never	55 (38.2)	41 (74.6)	9 (16.4)	5 (9.1)	p=0.5152
Ever	89 (61.8)	58 (65.2)	20 (22.5)	11 (12.4)	
Drinking status					
Never	84 (58.3)	58 (69.1)	17 (20.2)	9 (10.7)	p=1.000
Ever	60 (41.7)	41 (68.3)	12 (20.0)	7 (11.7)	
Cancer sub-site					
Tongue	78 (54.2)	55 (70.5)	16 (20.5)	7 (9.0)	p=0.6791
Floor of mouth	22 (15.3)	17 (77.3)	2 (9.1)	3 (13.4)	
Gingiva	15 (10.4)	9 (60.0)	3 (20. 0)	3 (20.0)	
Buccal mucosa	15 (10.4)	9 (60.0)	5 (33.3)	1 (6.7)	
Other	14 (9.7)	9 (64.3)	3 (21.4)	2 (14.3)	
pT					
T1-T2	99 (68.7)	66 (66.7)	20 (20.2)	13 (13.1)	p=0.5891
T3-T4	45 (31.3)	33 (73.3)	9 (20.0)	3 (6.7)	
pN					
N0	97 (67.4)	66 (68.0)	19 (19.6)	12 (12.4)	p=0.8553
N1-N3	47 (32.6)	33 (70.2)	10 (21.3)	4 (8.5)	
pStage					
I-II	75 (52.1)	49 (65.3)	16 (21.3)	10 (13.3)	p=0.5697
III-IV	69 (47.9)	50 (72.5)	13 (18.8)	6 (8.7)	
Grading^a^					
G1-G2	104 (74.8)	69 (66.4)	22 (21.2)	13 (12.5)	p=0.5676
G3	35 (25.2)	26 (74.3)	7 (20.0)	2 (5.7)	
RT					
No	93 (64.6)	61 (65.6)	20 (21.5)	12 (12.9)	p=0.5422
Yes	51 (35.4)	38 (74.5)	9 (17.7)	4 (7.8)	
CT					
No	124 (86.1)	84 (67.7)	26 (21.0)	14 (11.3)	p=0.9285
Yes	20 (13.9)	15 (75.0)	3 (15.0)	2 (10.0)	
Surgical margins					
Negative	123 (85.4)	86 (69.9)	23 (18.7)	14 (11.4)	p=0.5699
Close/Positive	21 (14.6)	13 (61.9)	6 (28.6)	2 (9.5)	
Extracapsular spread					
Negative	128 (88.9)	89 (69.5)	24 (18.8)	15 (11.7)	p=0.5367
Positive	16 (11.1)	10 (62.5)	5 (31.3)	1 (6.3)	
TERT-rs2853669^a^					
TT	54 (38.6)	31 (57.4)	12 (22.2)	11 (20.4)	**p=0.0243**
TC/CC	86 (61.4)	64 (74.4)	17 (19.8)	5 (5.8)	

a The sum does not add up to total because of missing values; RT, radiotherapy; CT, chemotherapy.

Bold values indicate p<0.05.

### *TERT* Promoter Status

The distribution of *TERT* promoter mutations according to socio-demographic and clinical characteristics of patients are shown in **Table 1**. In the overall cohort, the promoter of *TERT* harboured mutations in 45/144 cases (31.3%). The *TERT* -124 C>T mutation was more common (29/144, 20.1%) than -146 C>T (16/144, 11.1%). These two mutations occurred in a mutually exclusive manner and with a heterozygous genotype. No mutations were observed in any of 57 adjacent available analysed mucosal specimens, 16 of which were surrounding mutated tumours. There was no statistically significant difference among analysed parameters with regard to *TERT* promoter mutation rate. We also genotyped 140 of 144 patients of our cohort for the rs2853669 SNP at -245 bp. A total of 86 patients (61.4%) carried the minor C-variant allele, for which 16 patients were homozygous and 70 were heterozygous. Fifty-four patients (38.6%) had the T/T genotype. Notably, patients with *TERT* promoter mutated tumours had a higher prevalence of the T/T genotype than patients with unmutated *TERT* promoter (p=0.0243) (**Table 1**).

### Telomere Length

**Figure 1:**
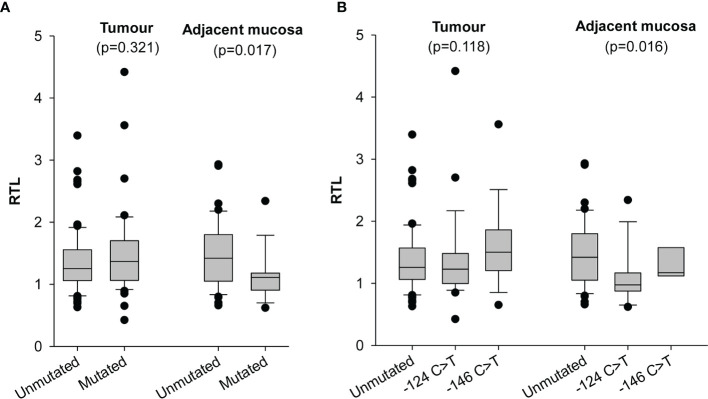
Distribution of relative telomere length (RTL) in tumour and adjacent mucosa according to *TERT* promoter status. **(A)** samples were stratified according to absence (Unmutated) and presence of -124 C>T or -146 C>T mutations (Mutated) in the *TERT* promoter region. **(B)** samples were stratified according to *TERT* promoter status in absence (Unmutated), presence of -124 C>T and presence of -146 C>T mutations in the *TERT* promoter region.

Measurement of RTL was obtained from 132 tumour tissues and 57 surrounding mucosal specimens. Values ranged between 0.42 and 4.42 (median 1.29) in tumours and between 0.62 and 2.93 (median 1.18) in surrounding mucosa; neither correlated with the age (data not shown). Telomere length in tumour cells and surrounding mucosa were not significantly associated with any of the measured demographic or clinical characteristics (**Supplementary Table 1**). In keeping with our previous findings (13), we found that the mucosa adjacent to tumours harbouring *TERT* promoter mutations had significantly shorter telomeres than those in adjacent mucosa of cancers with unmutated *TERT* promoter (p=0.017) (**Figure 1A**). In particular, the surrounding mucosa adjacent to tumours with -124 C>T mutated *TERT* promoter showed the shortest telomeres (p=0.016; **Figure 1B**), despite these patients being younger [median (IQR), 64(58–70) years] than those with unmutated tumours [median (IQR), 67(48–74) years] or with tumours harbouring -146 C>T mutations [median (IQR), 75(71–80) years] (p for age=0.065; data not shown). Conversely, telomere length in tumour tissue did not significantly differ according to the mutational status of *TERT* promoter (p=0.1182) (**Figures 1A, B**).

### Time-To-Event Analysis

The associations between socio-demographic and clinical characteristics of patients with clinical outcome are summarized in **Supplementary Table 2**. In a multivariate analysis adjusted for clinical variables (gender, age, pN, grading, surgical margins, and extracapsular invasion), it emerged that buccal mucosa sub-site, pathological lymph nodes, and G3 grading were significantly associated with increased risk of death (HR: 5.96, 95% CI: 1.16-30.73; p=0.0328; HR: 2.30, 95% CI: 1.12-4.75; p=0.0237; HR: 2.28, 95% CI: 1.14-4.56; p=0.0195; respectively).

**Figure 2:**
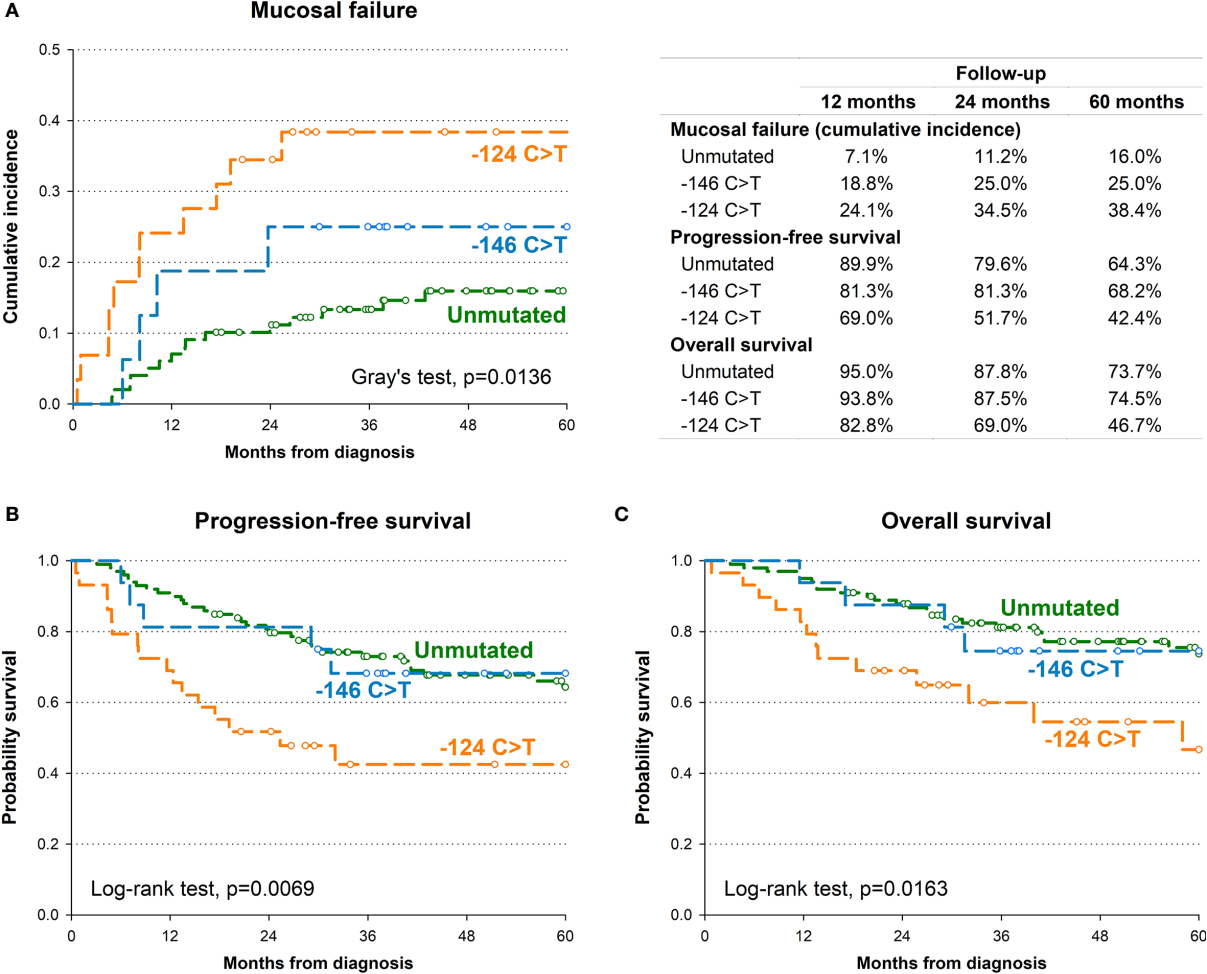
Kaplan-Meier estimates of cumulative incidence of mucosal recurrence **(A)**, progression-free survival **(B)** and overall survival **(C)** by *TERT* promoter.

In order to identify the potential impact of *TERT* promoter mutations on oncological outcome, we first investigated the association between *TERT* promoter status with PFS. Kaplan-Meier survival curve showed that the 5-year PFS for patients harbouring the -124 C>T mutation was 42.4% as opposed to 64.3% for patients without mutations, 68.2% for those harbouring the -146 C>T mutation (p=0.0069; **Figure 2B**). This association was confirmed by multivariate analysis (**Table 2**) after adjustment for clinical variables with a HR for progression of 2.71 (95% CI: 1.42-5.17; p=0.0024).

**Table 2:** Hazard ratio (HR) and corresponding 95% confidence interval (CI)a for mucosal failure, regional failure, progression, and death according to strata of TERT promoter status, rs2853669 genotype and telomere length.

	Pts	Mucosal failure			Regional failure			Progression			Death		
		n	HR (95% CI) ^b^	Wald χ^2^	n	HR (95% CI)	Wald χ^2^	n	HR (95% CI)	Wald χ^2^	n	HR (95% CI)	Wald χ^2^
*TERT* promoter													
unmutated	99	15	Ref		13	Ref		32	Ref		23	Ref	
-146C>T	16	4	2.46 (0.82-7.36)	p=0.1077	2	1.10 (0.21-5.73)	p=0.9127	5	1.35 (0.50-3.65)	p=0.5561	4	1.64 (0.53-5.08)	p=0.3891
-124C>T	29	11	2.75 (1.22-6.17)	**p=0.0143**	5	1.51 (0.48-4.73)	p=0.4822	16	2.71 (1.42-5.17)	**p=0.0024**	13	2.71 (1.30-5.66)	**p=0.0079**
													
TERT-rs2853669 (Dominant model)^c^													
CT/CC	86	15	Ref		12	Ref		25	Ref		18	Ref	
TT	54	14	1.36 (0.67-2.74)	p=0.3989	8	0.88 (0.37-2.11)	p=0.7820	28	1.80 (1.05-3.12)	**p=0.0343**	22	1.75 (0.93-3.29)	p=0.0837
													
TERT promoter – rs2853669													
unmut/-146C>T – CT/CC	69	10	Ref.		9	Ref.		19	Ref.		12	Ref.	
unmut/-146C>T – TT	42	8	1.33 (0.54-3.28)	p=0.5421	6	0.97 (0.35-2.69)	p=0.9476	18	1.50 (0.77-2.90)	p=0.2339	15	1.87 (0.86-4.08)	p=0.1156
-124C>T – CT/CC	17	5	2.69 (0.87-8.31)	p=0.0850	3	1.82 (0.47-7.06)	p=0.3857	6	1.79 (0.69-4.64)	p=0.2290	6	2.80 (0.98-8.00)	p=0.0539
-124C>T – TT	12	6	2.88 (1.01-8.25)	**p=0.0484**	2	1.06 (0.17-6.73)	p=0.9521	10	5.36 (2.30-12.48)	**p<0.0001**	7	4.05 (1.47-11.12)	**p=0.0067**
													
RTL (tumor)													
≤1.29^d^	66	15	Ref		8	Ref		25	Ref		21	Ref	
>1.29	66	13	0.92 (0.39-2.16)	p=0.8518	9	1.17 (0.36-3.87)	p=0.7946	23	1.04 (0.57-1.89)	p=0.8950	16	0.90 (0.45-1.78)	p=0.7564
													
RTL (surrounding mucosa)^e^													
>1.18^d^	26	3	Ref		3	Ref		5	Ref		3	Ref	
≤1.18	25	6	2.38 (0.48-11.73)	p=0.2869	1	0.07 (0.00-3.15)	p=0.1726	8	1.23 (0.37-4.14)	p=0.7366	8	2.21 (0.53-9.31)	p=0.2789

a Estimated from Cox proportional hazard model, adjusting for gender, age, pN, grading, surgical margins, and extracapsular invasion.

b Adjusted for competing risk according to Fine-Gray model;

c Results for the best genetic model on OS;

d Cut-off were defined according to median value;

e Analysis restricted to patients with negative surgical margins; Pts, patients; RTL, relative telomere length.

Bold values indicate p<0.05.

The presence of the -124 C>T mutation was also consistently associated with shorter OS, with 46.7% of patients alive after 5 years, in comparison to 73.7% and 74.5% of patients without mutations or harbouring the -146 C>T mutation, respectively (p=0.0163; **Figure 2C**). Multivariate analyses confirmed the negative effect of the -124 C>T mutation on prognosis, with a HR of death of 2.71 (95% CI: 1.30-5.66; p=0.0079) (**Table 2**). The negative impact of the -124 C>T mutation on clinical outcome was likely due to poorer mucosal control; indeed, based upon cumulative incidence estimates, patients with tumours harbouring this mutation suffered a 5-year mucosal failure rate of 38.4% in comparison to 16% and 25% in patients without mutations or harbouring the -146 C>T mutation, respectively (p=0.0136; **Figure 2A**). This association remained statistically significant in the multivariate analysis (HR: 2.75, 95% CI: 1.22-6.17; p=0.0143) (**Table 2**). These results suggest that the -124 C>T point mutation may be a risk factor for the aggressiveness of OCSCC compared to the -146 C>T mutation and unmutated *TERT* promoters which appear to be associated with a more favourable clinical outcome. Notably, the surrounding mucosa adjacent to tumours with -124 C>T mutated *TERT* promoter had the shortest telomeres (p=0.016; **Figure 1B**), and, in line with our previous studies (26, 27), adjacent mucosa with shorter telomeres (below the median value) showed a high, albeit not significant risk of tumour relapse (**Table 2**).

Kaplan-Meier survival analysis revealed that carriers of the T/T rs2853669 genotype showed significantly worse PFS (p=0.008) and OS (p=0.021) compared with C carriers (T/C+C/C genotypes) (**Supplementary Figure 1B, C**). The negative impact of the T/T genotype was confirmed in the multivariate analysis for progression (HR: 1.80, 95% CI: 1.05-3.12; p=0.0343) but not for death (HR: 1.75, 95% CI: 0.93-3.29; p=0.0837) (**Table 2**).

To evaluate if the SNP rs2853669 genotype can modulate the effect of *TERT* promoter mutations on oncological outcome, the potential role of the -124 C>T *TERT* promoter mutation as a prognostic parameter in OCSCC patients was assessed according to their rs2853669 background. Multivariate analysis revealed that the risk of mucosal failure (HR: 2.88, 95% CI: 1.01-8.25; p=0.0484), progression (HR: 5.36, 95% CI: 2.30-12.48; p<0.0001) and death (HR: 4.05, 95% CI: 1.47-11.12; p=0.0067) were significantly increased in patients with -124 C>T mutated tumours carrying the T/T genotype of the rs2853669 (**Table 2**) compared to patients without this mutation, and C carriers of the SNP.

## Discussion

In the present investigation, we observed that approximately one-third of OCSCC samples harboured *TERT* promoter mutations with the -124 C>T mutation having a significant adverse impact on the outcome; particularly, when coexisting with the T/T genotype of rs2853669, -124 C>T mutation increased the risk of death by 4 times.

In the literature, the frequency of *TERT* promoter mutations in OCSCC varies significantly among studies ranging from 30.4 to 75% (13, 28–34). This variability could be attributable to different patient population characteristics or methodological approaches. In our cohort, we found 31.3% (45 of 144) of OCSCC samples harboured *TERT* promoter mutations, which was in line with other studies (13, 28, 30, 33, 34). In agreement with other studies on OCSCC (20, 28–32), the two mutations have different frequency, with a higher prevalence of -124 C>T (29 of 144) compared to -146 C>T (16 of 144).

With respect to oncological outcomes, an important finding emerging from this study is that the two somatic *TERT* promoter mutations displayed different behaviour. Indeed, while patients with the -124 C>T *TERT* promoter mutation had a higher risk of mucosal failure and poorer DFS and OS, patients with tumours harbouring the -146 C>T mutation had an improved clinical outcome, similar to those with unmutated *TERT* promoter. The recruitment of the transcription factor GABPA, a member of ETS family, specifically to mutant *TERT* promoters mediates long-range chromatin interaction and enrichment of active histone marks, and hence drives *TERT* transcription (35). Although both the -124 C>T and -146 C>T mutations generate identical sequences, enable binding of GABPA transcription factors, and are equally efficient in increasing *TERT* transcription *in vitro* (36), previous reports demonstrated that these mutations are not functionally identical. Indeed, a peculiar pathway of activation by non-canonical NF-ĸB signalling was only described for the -146 C>T mutation (37, 38). In addition, *in vivo*, the -124 C>T mutation was associated with higher *TERT* expression/telomerase activity compared to -146 C>T (39, 40). A significant body of evidence has demonstrated that high levels of tumour TERT expression and/or telomerase activity are significantly associated with aggressiveness of disease, advanced clinical stage, and poor OS and/or DFS in several types of tumours, including UADT SCC (13, 26, 27, 41). The mechanism(s) by which high TERT expression ultimately facilitates cancer progression and constitutes a prognostic factor are not completely elucidated, and seems not be attributable only to TERT’s ability to maintain telomere length. Indeed, accumulating evidence suggests that TERT may also contribute to carcinogenesis *via* telomere length-independent mechanisms, including enhancement of proliferation, resistance to apoptosis, inflammation, invasion and metastasis altogether contributing towards a more aggressive phenotype of cancer cells (10, 11, 42–50). Therefore, it is conceivable that the -124 C>T *TERT* promoter mutation, inducing higher expression of TERT in the tumour, results in the increased severity of disease as we observed in our cohort of OCSCC patients. Corroborating our results, Arantes et al. (33) found that the -124 C>T *TERT* promoter mutation was associated with increased risk of tumour relapse and death in a cohort of 88 Brazilian patients with SCC of the UADT. However, other studies in different tumour types have reported contradicting clinical effects of *TERT* promoter mutations, ranging from poorer survival associated with the -146 C>T *TERT* promoter mutation to unchanged clinical outcome (28, 29, 32, 51–54). Given that the two mutations create an identical sequence corresponding to a *de novo* binding site for ETS transcription factors, these alternative results may depend on the genetic context, including the SNP background in which *TERT* mutations arise.

For the common polymorphism rs2853669 T>C, which disrupts a pre-existing ETS2 binding site within the *TERT* core promoter, controversial clinical impacts have been reported (12, 17–20). Our study demonstrates for the first time that the rs2853669 T/T genotype influences the clinical outcome of OCSCC patients, being significantly associated with increased risk of disease progression. Importantly, the coexistence of the T/T genotype of rs2853669 and the -124 C>T *TERT* promoter mutation is associated with a significantly poorer prognosis including mucosal failure, disease progression and death. The effect of the rs2853669 SNP may be related to higher telomerase activity and *TERT* expression conferred by the T/T genotype (15) that can also additionally intensify the transactivation activity of *TERT* promoter mutations (14). Thus, we can speculate that high *TERT* levels conferred by the -124 C>T *TERT* promoter mutation and/or rs2853669 T/T genotype may promote tumour progression, probably as a consequence of the extra-telomeric non-canonical functions of telomerase. Unfortunately, we did not have enough tumour material to contemporaneously analyse *TERT* promoter status and TERT expression/activity, and further studies should be undertaken to extend and validate these findings.

A secondary finding of our study was the absent of *TERT* promoter mutations in the matched adjacent mucosa. This partly differs from a previous study by Chang et al. (29), and may be due to the reduced number of adjacent normal mucosal specimens available in our cohort. Nonetheless, the finding that metastatic and recurrent head and neck squamous cell carcinomas have more *TERT* promoter mutations compared to primary tumours (31) suggests that the acquisition of these mutations is a late event in carcinogenesis, and may explain the lack of *TERT* promoter mutations in the tumour’s adjacent mucosa.

Interestingly, we confirmed that telomeres in mucosa adjacent to *TERT* promoter mutated tumours were significantly shorter than those adjacent to tumours retaining unmutated *TERT* promoter (13), and additionally we found that the mucosa adjacent to -124 C>T mutated tumours had the shortest telomeres. As critically short telomeres are a hallmark of genomic instability associated to carcinogenesis and may be considered a marker of field cancerization (26, 27), is not surprising that patients harbouring the -124 C>T *TERT* promoter mutation showed a significantly increased risk of tumour relapse. These data, for the first time, support a prognostic role for tumour relapse of the -124 C>T *TERT* promoter mutation in patients with OCSCC likely related to the very short telomeres in the mucosa surrounding the tumour in which the mutation arises.

In conclusion, we found that the -124 C>T *TERT* promoter mutation, as well as the T/T genotype of the rs2853669 SNP, may be a risk factor for the aggressiveness of OCSCC, and the coexistence of these genetic variations might represent a greater risk of adverse outcome. Supported by the fact that the clinical significance of this mutation is consistent with the biological properties of TERT, that *TERT* promoter mutations were found to stratify the prognosis in several other cancers, are easy to identify using tissue from routinely collected biopsies and address the unmet clinical need of having a validated prognostic marker for OCSCC, our observations raise the possibility that the -124 C>T *TERT* promoter mutation in combination with the SNP rs2853669 T/T genotype may serve as a valuable prognostic marker in this cancer, with the ability to guide therapeutic and follow-up strategies.

## Data Availability Statement

The raw data supporting the conclusions of this article will be made available by the authors, without undue reservation.

## Ethics Statement

The studies involving human participants were reviewed and approved by the Treviso/Belluno provinces (protocol code 346/AULSS9, March 10, 2015), Italy. The patients/participants provided their written informed consent to participate in this study.

## Author Contributions

Conceptualization: ADR, PBR. and SG. Methodology: SG. Statistical analysis: JP. Investigation: SG, PBR, ER, GT, LA, RDC, MR, PN, AM, VC, MT, LB, GS, EE, MMa, MS, RB, APDT, MG, MMo, and GE. Resources: ADR. Writing-original draft preparation: SG and PBR. Writing-review and editing: SG, PBR, JF, and ADR. Supervision: ADR. Project administration: PBR and SG. All authors contributed to the article and approved the submitted version.

## Conflict of Interest

The authors declare that the research was conducted in the absence of any commercial or financial relationships that could be construed as a potential conflict of interest.

## Publisher’s Note

All claims expressed in this article are solely those of the authors and do not necessarily represent those of their affiliated organizations, or those of the publisher, the editors and the reviewers. Any product that may be evaluated in this article, or claim that may be made by its manufacturer, is not guaranteed or endorsed by the publisher.
