# Supplementary material for: TERT Promoter Mutations and rs2853669 Polymorphism: Useful Markers for Clinical Outcome Stratification of Patients With Oral Cavity Squamous Cell Carcinoma
Source: Front Oncol. 2021 Nov 10;11:782658. doi: 10.3389/fonc.2021.782658 (PMC8631274; doi:10.3389/fonc.2021.782658)
Supplement: Supplementary file 1 [file DataSheet_1.pdf]

Supplementary Material

Supplementary Table 1. Relative telomere length (RTL) in tumour and surrounding mucosa of oral cavity squamous cell carcinoma patients according to socio-demographic and clinical characteristics.

	RTL tumour		RTL surrounding mucosa	
	**N (%)**	**Median (IQR)**	**N (%)**	**Median (IQR)**
Overall	132	1.29 (1.06-1.58)	57	1.18 (0.99-1.65)
Sex				
Female	58	1.29 (1.05-1.70)	24	1.48 (0.99-1.68)
Male	74	1.28 (1.07-1.51)	33	1.16 (1.04-1.42)
		p=0.4184		p=0.5606
Age (years)				
<60	48	1.30 (1.08-1.53)	19	1.16 (1.02-1.47)
60-69	36	1.24 (1.04-1.53)	18	1.38 (0.99-1.80)
≥70	48	1.32 (1.04-1.74)	20	1.19 (0.98-1.61)
		p=0.6531		p=0.7303
Smoking status				
Never	50	1.22 (1.04-1.46)	25	1.07 (0.99-1.38)
Ever	82	1.33 (1.07-1.63)	32	1.45 (1.13-1.78)
		p=0.2244		p=0.0703
Drinking status				
Never	79	1.34 (1.10-1.63)	33	1.20 (1.06-1.70)
Ever	53	1.20 (1.02-1.45)	24	1.12 (0.99-1.64)
		p=0.0713		p=0.4005
Cancer sub-site				
Tongue	71	1.31 (1.04-1.57)	39	1.18 (0.99-1.63)
Floor of mouth	20	1.15 (0.94-1.53)	7	1.63 (1.19-2.34)
Gingiva	14	1.25 (1.07-1.63)	5	1.17 (1.13-1.57)
Buccal mucosa	14	1.48 (1.31-1.93)	3	1.80 (0.99-2.09)
Other	13	1.20 (1.11-1.46)	3	1.06 (0.62-1.11)
		p=0.3006		p=0.1879
pT				
T1-T2	93	1.33 (1.06-1.59)	43	1.19 (0.99-1.70)
T3-T4	39	1.20 (1.07-1.49)	14	1.15 (0.80-1.38)
		p=0.2813		p=0.3937
pN				
N0	89	1.26 (1.06-1.53)	42	1.19 (0.99-1.63)
N1-N3	43	1.33 (1.07-1.68)	15	1.18 (1.02-1.88)
		p=0.7541		p=0.7305
pStage				
I-II	71	1.31 (1.06-1.59)	35	1.33 (0.99-1.65)
III-IV	61	1.26 (1.07-1.52)	22	1.15 (0.99-1.75)
		p=0.4764		p=0.4409

Grading[a]				
G1-G2	97	1.30 (1.06-1.57)	41	1.18 (0.99-1.57)
G3	30	1.25 (1.11-1.70)	13	1.13 (1.00-1.74)
		p=0.8226		p=0.9435
RT				
No	86	1.31 (1.04-1.57)	41	1.33 (0.99-1.70)
Yes	46	1.27 (1.08-1.68)	16	1.15 (1.02-1.38)
		p=0.8504		p=0.3464
CT				
No	115	1.31 (1.06-1.59)	51	1.18 (0.99-1.65)
Yes	17	1.17 (1.07-1.47)	6	1.13 (0.79-1.75)
		p=0.2652		p=0.5849
Surgical margins				
Negative	111	1.31 (1.07-1.59)	51	1.20 (0.99-1.70)
Close/Positive	21	1.17 (0.88-1.52)	6	1.16 (0.89-1.19)
		p=0.3687		p=0.3424
Extracapsular spread				
Negative	118	1.31 (1.08-1.57)	52	1.19 (0.99-1.64)
Positive	14	1.11 (0.88-1.68)	5	1.18 (1.13-1.75)
		p=0.4378		p=0.7670
TERT-rs2853669[a]				
TT	51	1.30 (1.10-1.51)	20	1.12 (0.98-1.36)
TC/CC	78	1.29 (1.06-1.67)	35	1.42 (0.99-1.87)
		p=0.6576		p=0.0799

RTL: Relative telomere length; IQR: interquartile range; p: probability for Fisher exact test; [a]the sum does not add up to total because of missing values; RT: radiotherapy; CT: chemotherapy.

Supplementary Table 2. Hazard ratio (HR) of death and corresponding 95% confidence interval (CI) according to socio-demographic and clinical characteristics.

	**N (%)**	**Deaths**	**HR (95% CI)[a]**	**Wald χ^2**
Sex				
Female	63 (43.8)	15	Ref.	
Male	81 (56.2)	25	1.23 (0.62-2.44)	p=0.5523
Age (years)				
<60	54 (37.5)	16	Ref.	
60-69	39 (27.1)	9	0.88 (0.38-2.03)	p=0.7599
≥70	51 (35.4)	15	1.45 (0.69-3.04)	p=0.3240
Smoking status				
Never	55 (38.2)	18	Ref.	
Ever	89 (61.8)	22	0.73 (0.36-1.50)	p=0.3953
Drinking status				
Never	84 (58.3)	19	Ref.	
Ever	60 (41.7)	21	1.66 (0.85-3.26)	p=0.1384
Cancer sub-site				
Tongue	78 (54.2)	19	1.65 (0.37-7.31)	p=0.5092
Floor of mouth	22 (15.3)	7	1.85 (0.37-9.36)	p=0.4569
Gingiva	15 (10.4)	2	Ref.	
Buccal mucosa	15 (10.4)	8	5.96 (1.16-30.73)	**p=0.0328**
Other	14 (9.7)	4	1.38 (0.24-8.00)	p=0.7189
pT				
T1-T2	99 (68.7)	24	Ref.	
T3-T4	45 (31.3)	16	1.17 (0.59-2.33)	p=0.6472
pN				
N0	97 (67.4)	19	Ref.	
N1-N3	47 (32.6)	21	2.30 (1.12-4.75)	**p=0.0237**
pStage				
I-II	75 (52.1)	13	Ref.	
III-IV	69 (47.9)	27	1.08 (0.40-2.95)	p=0.8762
Grading[b]				
G1-G2	104 (74.8)	23	Ref.	
G3	35 (25.2)	17	2.28 (1.14-4.56)	**p=0.0195**
RT				
No	93 (64.6)	19	Ref.	
Yes	51 (35.4)	21	0.98 (0.41-2.30)	p=0.9550
CT				
No	124 (86.1)	30	Ref.	
Yes	20 (13.9)	10	0.94 (0.39-2.27)	p=0.8968

Surgical margins				
Negative	123 (85.4)	30	Ref.	
Close/Positive	21 (14.6)	10	1.24 (0.76-2.03)	p=0.3907
Extracapsular spread				
Negative	128 (88.9)	32	Ref.	
Positive	16 (11.1)	8	0.96 (0.39-2.37)	p=0.9322

[a]Estimated from Cox proportional hazards model, adjusting for sex, age, pN, grading, surgical margins, and extracapsular invasion; [b]The sum does not add up to total because of missing values; RT: radiotherapy; CT: chemotherapy.

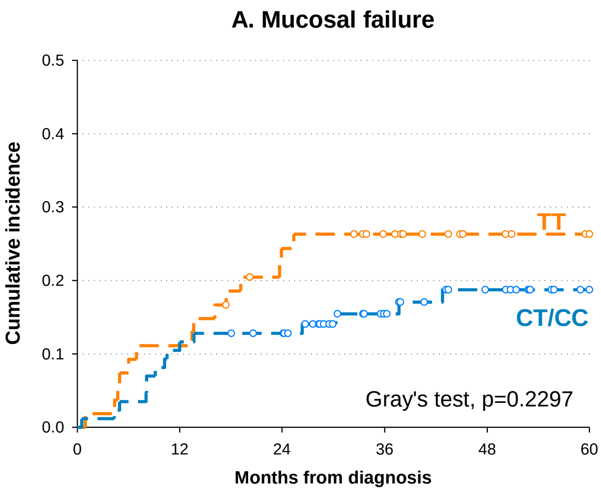

TERT-rs2853669	Follow-up		
	12 months	24 months	60 months
**Mucosal failure (cumulative incidence)**			
CT/CC	11.6%	12.8%	18.7%
TT	11.1%	24.4%	26.3%
**Progression-free survival**			
CT/CC	86.1%	79.0%	68.4%
TT	81.5%	64.6%	42.9%
**Overall survival**			
CT/CC	91.9%	86.0%	77.0%
TT	92.6%	79.4%	52.2%

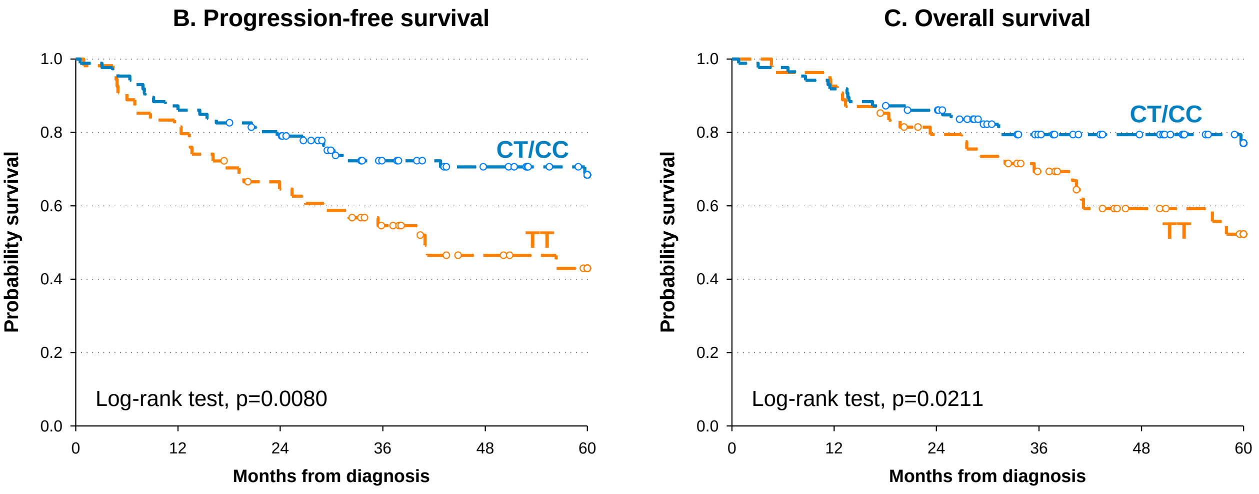

Supplementary Figure 1. Kaplan-Meier estimates of mucosal control **(A)**, progression-free survival **(B)** and overall survival **(C)** according to *TERT*-rs2853669 genotype.
